# Job Satisfaction of Fitness Professionals in Portugal: A Comparative Study of Gender, Age, Professional Experience, Professional Title, and Educational Qualifications

**DOI:** 10.3389/fpsyg.2020.621526

**Published:** 2021-01-13

**Authors:** Liliana Ricardo Ramos, Dulce Esteves, Isabel Vieira, Susana Franco, Vera Simões

**Affiliations:** ^1^Sport Sciences School of Rio Maior, Polytechnic Institute of Santarém, Santarém, Portugal; ^2^Department of Sports Sciences, University of Beira Interior, Covilhã, Portugal; ^3^Life Quality Research Centre (CIEQV), Santarém, Portugal; ^4^Research Centre in Sports Sciences, Health Sciences and Human Development (CIDESD), Vila Real, Portugal

**Keywords:** fitness, fitness professionals, job satisfaction, Job Satisfaction Scale, comparative study

## Abstract

This research characterizes and compares the job satisfaction of fitness professionals in Portugal between genders, ages, professional experience, professional title, and educational qualifications. A total of 401 fitness professionals answered the online questionnaire Job Satisfaction Scale, which has 16 factors rated on a Likert scale with seven levels. The statistical analysis comprises descriptive and statistical tests to compare the results of two (*t*-test) or more (ANOVA) groups. Overall, the results demonstrated that fitness professionals were moderately satisfied with their work. The lower degrees of job satisfaction were concerning salary, opportunities for promotion, and stability at work. The highest levels of job satisfaction were related to the freedom to choose their work method, their work colleagues, the physical conditions of their workplace, and the opportunity to use their competencies. There were no differences in job satisfaction in terms of gender and a few differences between professional title and between educational levels. Regarding age and professional experience, the results showed significant differences in almost all factors, suggesting that, as fitness professionals get older and more experienced, their job satisfaction is greater. The results of this study suggest that even though fitness professionals are, in general, moderately satisfied with their work, some aspects can be improved by employers to increase their satisfaction levels. Such findings would assist employers in guiding the job satisfaction of their employees with consequent enhancements in the services provided by fitness professionals, which can increase participants’ retention in exercise fitness activities.

## Introduction

The fitness market has increased exponentially in recent decades, and in 2019, there were nearly 210,000 gyms/health clubs worldwide with approximately 184 million clients and a total income of US $96.7 billion (International Health Racquet and Sportsclub Association, 2020). In Europe, there were 64.8 million fitness clients in 2019, attending almost 63,644 gyms/health clubs and generating incomes of around €28.2 billion (EuropeActive, 2020). Most of these European fitness clients stemmed from Germany, the United Kingdom, France, Spain, and Italy. These five countries represent 65% of the total European income in the fitness industry.

Portugal is no exception to the rise in the fitness industry; in 2019, nearly 688,210 fitness clients were attending 1,100 gyms/health clubs in Portugal [Association of Gyms and Academies of Portugal—PortugalActive (AGAP, 2020)]. The number of fitness professionals has grown, and in 2018, the Portuguese Institute of Sport and Youth (IPDJ, 2018) approximated that there were 14,000 fitness professionals in Portugal with a valid professional title, among which are technical directors (DTs) and physical exercise instructors (TEFs). In 2019, nearly 12,000 collaborators were working as fitness professionals in Portugal (AGAP, 2020). Fitness professionals’ importance for the satisfaction and retention of clients has been reported in several studies to be essential for the growth of the sector (Papadimitriou and Karteroliotis, 2000; Murray and Howat, 2002; Makover, 2003; Theodorakis et al., 2004; Tsitskari et al., 2014; Campos et al., 2016).

Job satisfaction can be described as a person’s general perception of various aspects of the work that they develop (Spector, 1997). Another more specific definition of job satisfaction relates to how the person feels about their work in the context of their previous experiences, expectations, and/or alternatives that are presented to them (Balzer et al., 1990). High levels of satisfaction at work may contribute to a person’s healthy emotional and mental condition, thereby resulting in a positive attitude to the organization. On the other hand, a lack of satisfaction may result in an employee’s worse performance and negatively affect the organization (Aziri, 2011).

Some issues inherent to fitness professional occupation seem to affect, in some studies, their job satisfaction as well as other factors, such as burnout and the intention to leave the organization. Issues related to excessive hours of physical exercise have been identified as one of the problems associated to these professionals, resulting in injuries and/or musculoskeletal pain (Malliou et al., 2014; Bratland-Sanda et al., 2015). The job stability is also an issue and revealed a positive correlation with job satisfaction in fitness professionals from Greece and Spain (Koustelios et al., 2003; Gil, 2013). The hours of work and the range of the working time, sometimes extended from the opening hours (e.g., 7 AM) to closing (e.g., 10 PM), can also be a problem for fitness professionals (Franco, 2020). Job satisfaction was also associated, in fitness professionals, with job security, absenteeism, productivity, and the business’s turnover (Koustelios et al., 2003), as well as its organizational culture (Macintosh and Doherty, 2010), organizational commitment (Culibrk et al., 2018), staff burnout (Gil, 2013), and an employee’s autonomy at work (Terason, 2018; Sawang et al., 2020), among other factors.

This subject has already been the target of several studies, some of which have compared also sociodemographic and other characteristics that can affect fitness professionals’ levels of job satisfaction. In Spain, a study with 631 fitness professionals found that its sample presented moderate levels of satisfaction at work, with no relationship found between their satisfaction levels and participants’ gender or professional experience. The fitness professionals with higher ages and fewer educational qualifications were found to be more satisfied at work (Bernabé et al., 2017). In Brazil, a study with 497 fitness professionals verified that most of these professionals (88.9%) were satisfied at work (Bevilacqua et al., 2014). A Canadian study with 416 fitness professionals investigated the degree to which the organizational culture can influence an employee’s satisfaction at work, along with their intention of leaving the organization. The organizational culture was found to justify 14.3% of the variance in their levels of satisfaction, while it influenced 50.5% of the professionals in their decision to leave the organization (Macintosh and Doherty, 2010). In Greece, a study into professional security and satisfaction at work investigated 97 fitness professionals and demonstrated a correlation between these two variables (Koustelios et al., 2003). A Thai study explored the influence of autonomy at work upon job satisfaction, finding that an employee’s autonomy when performing tasks—mainly those that demanded more responsibility—was associated with greater professional satisfaction (Terason, 2018). In Portugal, a study from Oliveira (2017) was focused upon understanding the factors that promoted professional satisfaction for 53 fitness professionals of gyms/health clubs. The professional satisfaction of the managers/supervisors was found to be more influenced by the exercise programs offered by their organization (specifically if the programs were innovating), whereas their subordinate colleagues referred to the environment within the organization and the degree of sympathy between staff and clients as the most important factors linked to their job satisfaction.

However, when it comes to the job satisfaction of fitness professionals in Portugal with a professional title of TEF or DT, not much information was found. Taking into consideration the importance of these professionals within the industry, there is a gap in the existing research. Findings related to TEFs and DTs would be beneficial for identifying the critical factors of job satisfaction in those professionals. Higher levels of job satisfaction can prevent dropout and result in better service, and better service, in consequence, provide superior client satisfaction and retention, increasing the number of people that practice exercise and contributing to lower levels of physical inactivity in Portugal (European Opinion Research Group, and Special Eurobarometer 472, 2018).

Therefore, the aims of this study are to (1) verify the levels of job satisfaction of the fitness professionals in Portugal and (2) analyze and compare the fitness professionals’ job satisfaction levels concerning gender, age, professional experience, professional title, and educational qualification.

## Materials and Methods

### Sample

The sample comprised 401 individuals working as fitness professionals (Table 1), 58.1% with the professional title of TEF and 41.9% with the professional title of DT. Regarding their gender, 50.6% of participants were female and 49.4% were male. The average age of the sample was 30.6 ± 7.6 years [mean (*M*) ± standard deviation (SD)]. Most of the fitness professionals were between 18 and 29 years (55.9%), followed by the group of 30–44 years (37.9%), and the smallest group were between 45 and 65 years (6.2%). The average professional experience was 8.9 ± 6.6 years (*M* ± SD), with 63.8% having less than 10 years of professional experience and 35.2% equal or more than 10 years. When it came to their educational qualifications, most of the fitness professionals (86.3%) had a bachelor’s degree or higher qualification (i.e., a master’s degree and/or a Ph.D.), and 13.7% have a high school level.

**TABLE 1:** Frequencies of the variables (*n* = 401 fitness professionals).

Gender	Frequency (%)
Female	50.6
Male	49.4
Age	
Group 1: 18–29	55.9
Group 2: 30–44	37.9
Group 3: 45–65	6.2
Educational qualifications	
Secondary school	13.7
Higher degree	86.3
Professional title	
TEF	58.1
DT	41.9
Professional experience	
<10 years	63.8
≥10 years	35.2

### Instruments and Procedures

Job Satisfaction Scale (Warr et al., 1979), translated and validated to Portuguese (Ramos et al., 2020), was employed to measure job satisfaction of the fitness professionals. The Job Satisfaction Scale is composed of 15 items based on various aspects of job satisfaction, along with a 16-item referring to job satisfaction as a whole. In the validation of Job Satisfaction Scale (JSS), the confirmatory analyses revealed that a unidimensional model (1 factor/16 items) exposed a best model adequacy coefficient (Ramos et al., 2020), so the results were analyzed considering this model. The respondents had a Likert scale with seven levels of response for each factor, ranging between 1 (extremely dissatisfied) and 7 (extremely satisfied). In addition to the JSS, questions of sociodemographic characterization were carried out. The questionnaire was conducted on an online platform, SurveyMonkey, between November 2019 and March 2020, before the coronavirus disease 2019 (COVID-19) pandemic. The questionnaire was disseminated through social networks, higher education institutions, training providers, fitness sector associations, as well as at fitness events and conventions. Ethical approval was obtained from the ethics and scientific board of the University of Beira-Interior, Portugal.

### Statistical Analysis

First, descriptive statistics were conducted upon the measures of central tendency (mean) and dispersion (standard deviation) for all variables of the Job Satisfaction Scale. *t-*tests with independent sampling (comparing the means of two groups) were then carried out to verify the differences in job satisfaction concerning genders, educational qualifications, professional titles, and professional experiences. To compare the average numbers in more than two groups (in relation with age), an *ANOVA* (*F-*test) was employed, complemented with a *post hoc* Tukey’s test (if the variances were found to be homogeneous according to a Levene’s test) or a *post hoc* Games–Howell test (if the variances were not homogeneous) (Ho, 2014). The level of significance adopted was *p* < 0.05. All tests were conducted using SPSS 26.0.

## Results

The results demonstrated that the job satisfaction of fitness professionals, based on the average of every factor in the study, was 4.88 on a scale of 1–7. This corresponds to “moderately satisfied” (Table 2). Through analysis, it was possible to verify that the fitness professionals held lower degrees of satisfaction concerning their salary, with satisfaction levels of 4.1 ± 1.5 (*M* ± SD), their opportunities for promotion (4.2 ± 1.6), and their stability at work (4.3 ± 1). These three factors all registered a level close to 4, which corresponds to “neither satisfied nor unsatisfied.” On the other hand, the factors for which the fitness professionals held the highest levels of professional satisfaction were the freedom to choose their work method (5.6 ± 1.4), their work colleagues (5.6 ± 1.2), the physical conditions of their workplace (5.3 ± 1.1), and the opportunity to use their competencies (5.3 ± 1.3).

**TABLE 2:** General job satisfaction and comparison by age and professional experience.

Factors	General (*M* ± *SD*)	Age (*M* ± *SD*)			Age comparison	Professional experience (*M* ± *SD*)		Professional experience comparison
					
		Group 1 18–29 years	Group 2 30–44 years	Group 3 45–65 years	*ANOVA (F-test)*	<10 years	≥10 years	*T-test*
1. The physical work conditions	5.3 ± 1.2	5.2 ± 1.2	5.4 ± 1.2	5.7 ± 1.1	0.09	5.2 ± 1.3	5.6 ± 1.1	0.01*
2. The freedom to choose your own method of working	5.6 ± 1.4	5.4 ± 1.5	5.7 ± 1.4	6.2 ± 0.7	0.01*^2,3^	5.4 ± 1.5	5.8 ± 1.3	0.03*
3. Your fellow workers	5.6 ± 1.2	5.6 ± 1.2	5.6 ± 1.1	5.8 ± 0.8	0.62	5.6 ± 1.2	5.7 ± 1.1	0.33
4. The recognition you get for a good work	4.9 ± 1.5	4.8 ± 1.5	4.9 ± 1.5	5.7 ± 1.1	0.01*^2,3^	4.8 ± 1.5	5.1 ± 1.5	0.03*
5. Your immediate boss	4.9 ± 1.6	4.8 ± 1.6	5.0 ± 1.6	5.6 ± 1.1	0.04*^2,3^	4.8 ± 1.6	5.1 ± 1.5	0.04*
6. The amount of responsibility you are given	5.2 ± 1.3	5.1 ± 1.3	5.2 ± 1.4	6.0 ± 1.2	0.01*^2,3^	5.2 ± 1.3	5.4 ± 1.4	0.02*
7. Your rate of pay	4.1 ± 1.5	3.9 ± 1.5	4.4 ± 1.5	4.4 ± 1.7	0.01*^1^	3.9 ± 1.5	4.5 ± 1.4	0.01*
8. The opportunity to use your abilities	5.3 ± 1.3	5.2 ± 1.4	5.3 ± 1.3	5.9 ± 1.1	0.02*^2^	5.1 ± 1.4	5.6 ± 1.2	0.01*
9. The relations between management and workers in your organization	5.0 ± 1.5	4.9 ± 1.5	5.1 ± 1.4	5.3 ± 1.1	0.17	4.9 ± 1.5	5.3 ± 1.3	0.01*
10. Your chance of promotion	4.2 ± 1.6	4.0 ± 1.6	4.5 ± 1.5	4.7 ± 1.5	0.01*^1^	4.0 ± 1.6	4.7 ± 1.5	0.01*
11. The way your organization is managed	4.6 ± 1.5	4.5 ± 1.5	4.7 ± 1.5	5.4 ± 1.2	0.03*^2^	4.4 ± 1.5	5.0 ± 1.4	0.01*
12. The attention paid to suggestions you make	4.7 ± 1.4	4.6 ± 1.5	4.9 ± 1.4	5.0 ± 1.4	0.08	4.5 ± 1.5	5.1 ± 1.3	0.01*
13. Your hours of work	4.5 ± 1.5	4.3 ± 1.5	4.6 ± 1.6	5.2 ± 1.3	0.01*^2^	4.4 ± 1.5	4.6 ± 1.5	0.11
14. The amount of variety in your job	5.1 ± 1.3	5.0 ± 1.2	5.2 ± 1.3	5.3 ± 1.1	0.25	5.0 ± 1.3	5.3 ± 1.2	0.01*
15. Your job security	4.3 ± 1.8	4.1 ± 1.7	4.5 ± 1.8	4.8 ± 1.4	0.06	4.0 ± 1.7	4.7 ± 1.7	0.01*
16. Now, taking everything into consideration, how do you feel about your job as a whole?	5.1 ± 1.3	4.9 ± 1.3	5.1 ± 1.3	5.6 ± 1.1	0.03*^2^	4.9 ± 1.3	5.4 ± 1.3	0.00*
Mean	4.88	4.77	5.00	5.41		4.76	5.18	

*1, extremely dissatisfied; 2, very dissatisfied; 3, moderately dissatisfied; 4, neither satisfied nor dissatisfied; 5, moderately satisfied; 6, very satisfied; 7, extremely satisfied.*

*^∗^*p* ≤ 0.05. ^1^Differences between groups 1 and 2.*

*^2^Differences between groups 1 and 3.*

*^3^Differences between groups 2 and 3.*

Concerning their age (Table 2), it was verified that the older fitness professionals were most satisfied, in general, with their career. In all factors as age increased, job satisfaction improved, with significant differences between age groups in 10 of the 16 factors analyzed.

Regarding their professional experiences (Table 2), job satisfaction was generally found to be higher among fitness professionals with 10 or more years of professional experience; there were significant differences between professional experience levels in 14 of the 16 job satisfaction factors analyzed.

There were no differences between genders (Table 3) in any job satisfaction factors, and the values obtained by each gender (female and male) were very similar.

**TABLE 3:** Comparison of job satisfaction by gender, professional title, and academic qualifications.

Factors	Gender		Gender	Professional title		Professional	Educational qualifications		Educational qualifications
	(*M* ± *SD*)		comparison	(*M* ± *SD*)		title comparison	(*M* ± *SD*)		comparison
	Female	Male	*T-test*	TEF	DT	*T-test*	Secondary	Higher	*T-test*
							school	degree	
1. The physical work conditions	5.3 ± 1.2	5.3 ± 1.3	0.92	5.2 ± 1.2	5.4 ± 1.2	0.16	5.1 ± 1.3	5.3 ± 1.2	0.17
2. The freedom to choose your own method of working	5.5 ± 1.4	5.6 ± 1.4	0.21	5.6 ± 1.4	5.6 ± 1.5	0.97	5.6 ± 1.5	5.6 ± 1.4	0.97
3. Your fellow workers	5.7 ± 1.1	5.6 ± 1.2	0.31	5.7 ± 1.1	5.6 ± 1.3	0.47	5.5 ± 1.2	5.6 ± 1.1	0.55
4. The recognition you get for a good work	4.8 ± 1.4	4.9 ± 1.6	0.57	4.9 ± 1.5	4.9 ± 1.6	0.97	5.1 ± 1.5	4.8 ± 1.5	0.27
5. Your immediate boss	5.0 ± 1.5	4.9 ± 1.7	0.42	4.9 ± 1.6	5.0 ± 1.5	0.87	4.8 ± 1.5	4.8 ± 1.8	0.43
6. The amount of responsibility you are given	5.2 ± 1.2	5.2 ± 1.4	0.91	5.2 ± 1.3	5.2 ± 1.4	0.77	5.0 ± 1.6	5.2 ± 1.3	0.25
7. Your rate of pay	4.1 ± 1.5	4.1 ± 1.5	0.70	4.0 ± 1.6	4.2 ± 1.5	0.09	3.8 ± 1.7	4.1 ± 1.5	0.11
8. The opportunity to use your abilities	5.2 ± 1.2	5.3 ± 1.5	0.33	5.2 ± 1.4	5.3 ± 1.3	0.53	5.0 ± 1.7	5.3 ± 1.3	0.13
9. The relations between management and workers in your organization	5.1 ± 1.3	4.9 ± 1.6	0.14	4.9 ± 1.4	5.1 ± 1.5	0.34	4.7 ± 1.6	5.0 ± 1.4	0.15
10. Your chance of promotion	4.2 ± 1.4	4.2 ± 1.8	0.93	4.0 ± 1.6	4.5 ± 1.6	0.01*	3.9 ± 1.7	4.3 ± 1.6	0.13
11. The way your organization is managed	4.6 ± 1.4	4.6 ± 1.6	0.93	4.5 ± 1.5	4.8 ± 1.5	0.12	4.5 ± 1.6	4.7 ± 1.5	0.49
12. The attention paid to suggestions you make	4.8 ± 1.3	4.7 ± 1.6	0.51	4.6 ± 1.4	4.9 ± 1.5	0.10	4.4 ± 1.6	4.8 ± 1.4	0.01*
13. Your hours of work	4.6 ± 1.5	4.4 ± 1.6	0.27	4.5 ± 1.5	4.5 ± 1.5	0.91	4.5 ± 1.4	4.5 ± 1.5	0.10
14. The amount of variety in your job	5.1 ± 1.2	5.1 ± 1.3	0.93	5.1 ± 1.4	5.1 ± 1.4	0.73	3.9 ± 1.9	4.4 ± 1.7	0.23
15. Your job security	4.4 ± 1.7	4.2 ± 1.8	0.44	4.1 ± 1.8	4.6 ± 1.7	0.01*	3.9 ± 1.9	4.4 ± 1.7	0.11
16. Now, taking everything into consideration, how do you feel about your job as a whole?	5.0 ± 1.2	5.1 ± 1.4	0.59	5.0 ± 1.3	5.1 ± 1.4	0.36	4.8 ± 1.5	5.1 ± 1.3	0.12
Mean	4.91	4.88		4.84	4.99		4.66	4.87	

*1, extremely dissatisfied; 2, very dissatisfied; 3, moderately dissatisfied; 4, neither satisfied nor dissatisfied; 5, moderately satisfied; 6, very satisfied; 7, extremely satisfied. ^∗^*p* ≤ 0.05.*

Concerning their professional title (Table 3), the fitness professionals with a DT title demonstrated, on average, slightly higher levels of job satisfaction than those with the TEF title, but significant differences only were found in 2 of the 16 job satisfaction factors, namely, in the factors opportunities for promotion and stability at work.

Regarding their educational qualifications, fitness professionals who did not have a degree were compared with professionals that had a bachelor’s or higher degree (i.e., a master’s degree and/or a Ph.D.). The professionals with a higher education degree exhibited slightly higher values of job satisfaction, in 12 of the 16 factors, although this difference was significant only in the factor attention paid to the suggestions that they make at work.

## Discussion

The results of this study demonstrate that when it comes to their job satisfaction, the Portuguese fitness professionals present an average job satisfaction of 4.88, which corresponds to “moderately satisfied.” This finding is comparable with similar studies that have found fitness professionals to be “satisfied” with their work (Bevilacqua et al., 2014; Bernabé et al., 2017). Based on an analysis of factors that affect job satisfaction, this study concludes that those who present higher levels of professional satisfaction were concerning the freedom to choose their work methodology (5.6) and regarding their work colleagues (5.6). These results correspond with others previously obtained for Spanish fitness professionals (Gonzelez et al., 2016; Bernabé et al., 2017), in which a higher degree of satisfaction at work has been related to work colleagues. On the other hand, the lowest levels of professional satisfaction were found in salary (4.1) and opportunities for promotion (4.2). The lower levels of job satisfaction in salary are in accordance with several studies from other countries such as Spain (Koustelios et al., 2003; Gonzelez et al., 2016; Bernabé et al., 2017), Greece, and Brazil (Bevilacqua et al., 2014). The low satisfaction at work in the item low opportunities for promotion has also been evidenced in other studies (Bernabé et al., 2017; Grimaldi-Puyana et al., 2018). It can be suggested that the lack of a proper career path for a fitness professional in Portugal, with career progression and with salaries that increase under experience or educational qualifications, could contribute to low levels of job satisfaction in the item opportunities for promotion (Franco, 2020).

Concerning levels of job satisfaction and fitness professionals age groups, this study found that professionals with higher ages (i.e., between the ages 45 and 65) are more satisfied in all factors, being significantly different in 10 of the 16 factors, namely, the freedom to choose their work methods, the recognition of their good performance, their direct leadership, the amount of responsibility they are given, their salary, the opportunities to use their competencies, the way the organization is managed, their work schedule, and the work as a whole. Many of these factors are related to autonomy and the possibility for older professionals to work in the way that they intend with a higher degree of recognition and remuneration and a greater possibility of promotion. A study in Spain (Bernabé et al., 2017) also compare the age with job satisfaction of fitness professionals, verifying that there are differences in job satisfaction between professionals’ ages. However, the findings of that study were not linear; the higher indices of job satisfaction were presented by professionals aged between 60 and 70 and 16 and 29. In that sense, the findings relating to age were different from this study, specifically in the job satisfaction of the younger professionals.

Regarding professional experience, as with age, the professionals in this study with 10 or more years of experience demonstrated higher levels of job satisfaction than their less experienced counterparts in every factor, with significant differences in 14 of the 16 factors. Another study that investigated this variable, conducted in Spain, did not find a significant difference in job satisfaction between different professional experience levels (Bernabé et al., 2017). Our results seem to indicate that, as the fitness professional has more experience, he has higher satisfaction levels in work autonomy, being able to use his skills and obtaining greater recognition for that, financial or related to, for example, career promotion. Those factors probably increase with job experience, which can result in greater job satisfaction.

When comparing job satisfaction between genders, it was verified there is no significant difference between genders. These results are in concordance with an existing Spanish study (Bernabé et al., 2017), although in a Brazilian study (Anversa et al., 2019), female fitness professionals presented lower levels of satisfaction than their male counterparts in some physiological needs (like sleeping and eating) and their safety at work. In a study made in Marocco, with 171 employees from a fitness company, no significant difference has been found in job satisfaction levels between genders (Göksu and Keskin, 2018).

Concerning professional titles, there is a significant difference only in the factor opportunities for promotion and in job stability; for both factors, DTs have higher job satisfaction compared to TEFs. It is suggested that this difference in job satisfaction reflects a difficulty for the TEF to ascend to the position of DT in a gym/health club, and Portuguese Law, 39/2012 only authorizes professionals with the title of DT to exercise this position, which implies having a higher educational level in sport/exercise, generally more connected to coordination/supervision tasks and seen as a career promotion.

Lastly, this study compares job satisfaction between different educational qualifications. The results of these two groups show that fitness professionals with higher qualifications have slightly higher values of satisfaction in 12 of the 16 factors analyzed, but the study only found a significant difference with regards to the factor attention given to the suggestions that fitness professionals make. In this regard, graduates are significantly more satisfied than those with only have high school qualifications or less. It seems that employers value more the opinion of the most qualified fitness professionals. This finding contrasts with a study on fitness professionals in Spain (Bernabé et al., 2017), which found that those with fewer qualifications were more satisfied, while those with a degree were less satisfied.

The limitations presented in this study were related mainly to the representativeness of the population that was intended to be studied. Even with all the dissemination of the study, stopped by the COVID-19 pandemic, the sample was obtained by convenience, not allowing to represent statistically the studied population.

More studies are needed to further explore the levels of job satisfaction of fitness professionals in Portugal, incorporating additional factors such as quality of life and burnout. It would also be interesting to compare job satisfaction concerning the type of place where professionals provide services (health club, fitness boutique, fitness club, gym belonging to a chain, or an individual gym) as well as knowing and comparing the organizational environment of employers and their job satisfaction. These variables were explored together with job satisfaction in studies about fitness professionals in other countries (Gil, 2013; Bevilacqua et al., 2014), and it would be interesting to see if there is a relationship between them in Portugal. It could also be interesting to understand the employers’ opinion concerning aspects related to their employees’ job satisfaction, checking if there are differences in the perception of employers and employees. Another study that would be interesting to carry out would be to understand if the pandemic caused by COVID-19 and the serious consequences it caused in the fitness industry and the professionals of the sector affected the job satisfaction of these professionals and in what factors it did.

## Conclusion

The results showed that fitness professionals in Portugal are moderately satisfied with their work, with no differences in satisfaction in terms of gender and a few differences between professional title and between educational levels. Regarding age and professional experience, the results showed significant differences in almost all factors, suggesting that as professionals get older and more experienced, their job satisfaction increase.

It is possible that, throughout their careers, fitness professionals with the lowest levels of job satisfaction have drop out of the profession. An understanding of job satisfaction can help employers manage the satisfaction of their employees, preventing the dropout of fitness professionals and providing a better service made by fitness professionals, with positive consequences for the growth of the fitness industry.

## Data Availability Statement

The raw data supporting the conclusions of this article will be made available by the authors, without undue reservation.

## Ethics Statement

The studies involving human participants were reviewed and approved by the University of Beira Interior n°CE-UBI-Pk-2019-006:ID1126. The patients/participants provided their written informed consent to participate in this study.

## Author Contributions

LR wrote the sections of the manuscript. All authors contributed to the conception and design of the study, manuscript revision, and read and approved the submitted version.

## Conflict of Interest

The authors declare that the research was conducted in the absence of any commercial or financial relationships that could be construed as a potential conflict of interest.
